# Exploring the current landscape of single‐cell RNA sequencing applications in gastric cancer research

**DOI:** 10.1111/jcmm.18159

**Published:** 2024-03-17

**Authors:** Wireko Andrew Awuah, Sakshi Roy, Joecelyn Kirani Tan, Favour Tope Adebusoye, Zekai Qiang, Tomas Ferreira, Arjun Ahluwalia, Vallabh Shet, Amanda Leong Weng Yee, Toufik Abdul‐Rahman, Marios Papadakis

**Affiliations:** ^1^ Faculty of Medicine Sumy State University Sumy Ukraine; ^2^ School of Medicine Queen's University Belfast Belfast UK; ^3^ Faculty of Medicine University of St Andrews St Andrews UK; ^4^ Department of Oncology & Metabolism The University of Sheffield Sheffield UK; ^5^ Department of Clinical Neurosciences, School of Clinical Medicine University of Cambridge Cambridge UK; ^6^ Faculty of Medicine Bangalore Medical College and Research Institute Bangalore Karnataka India; ^7^ Faculty of Medicine University of Malaya Kuala Lumpur Malaysia; ^8^ Department of Surgery II, University Hospital Witten‐Herdecke University of Witten‐Herdecke Wuppertal Germany

**Keywords:** gastric cancer, gene expression profiling, medical oncology, molecular medicine, scRNA‐seq, single‐cell RNA sequencing, therapeutic targets, tumour heterogeneity, tumour microenvironment

## Abstract

Gastric cancer (GC) represents a major global health burden and is responsible for a significant number of cancer‐related fatalities. Its complex nature, characterized by heterogeneity and aggressive behaviour, poses considerable challenges for effective diagnosis and treatment. Single‐cell RNA sequencing (scRNA‐seq) has emerged as an important technique, offering unprecedented precision and depth in gene expression profiling at the cellular level. By facilitating the identification of distinct cell populations, rare cells and dynamic transcriptional changes within GC, scRNA‐seq has yielded valuable insights into tumour progression and potential therapeutic targets. Moreover, this technology has significantly improved our comprehension of the tumour microenvironment (TME) and its intricate interplay with immune cells, thereby opening avenues for targeted therapeutic strategies. Nonetheless, certain obstacles, including tumour heterogeneity and technical limitations, persist in the field. Current endeavours are dedicated to refining protocols and computational tools to surmount these challenges. In this narrative review, we explore the significance of scRNA‐seq in GC, emphasizing its advantages, challenges and potential applications in unravelling tumour heterogeneity and identifying promising therapeutic targets. Additionally, we discuss recent developments, ongoing efforts to overcome these challenges, and future prospects. Although further enhancements are required, scRNA‐seq has already provided valuable insights into GC and holds promise for advancing biomedical research and clinical practice.

## 1 BACKGROUND

Gastric cancer (GC) is a complex disease influenced by a diverse range of environmental and genetic factors. It stands as the third leading cause of cancer‐related mortality worldwide, accounting for approximately one out of every 12 cancer‐related deaths.^1^ Additionally, GC ranks fifth for cancer incidence, representing 5.7% of all newly diagnosed cases.^1^ Given its heterogeneous nature and marked aggressiveness, GC poses a significant global health challenge. Alternative preventive measures, such as dietary considerations, early diagnosis and appropriate treatments, have shown effectiveness in reducing the incidence of GC.^2^

To confront the heterogeneity and aggressiveness of GC, the emergence of single‐cell ribonucleic acid sequencing (scRNA‐seq) has introduced promising technology for profiling gene expression at the individual cell level.^3^, ^4^, ^5^ This transformative technique has revolutionized transcriptome analysis by providing unprecedented precision and depth, enabling a comprehensive understanding of cellular diversity within complex gastric tumour tissues. Through its capacity to unravel cellular heterogeneity, gene expression dynamics and intercellular interactions, scRNA‐seq has shed light on intricate biological processes, including cancer development and progression.

Notably, scRNA‐seq has facilitated the identification and characterization of distinct cell populations within gastric tumours, elucidating their molecular profiles and functions. Leveraging scRNA‐seq, studies conducted by Wang et al. (2021), and Zhou et al. (2023) have unveiled unique subtypes and cell states within GC, improving our understanding of tumour composition and highlighting potential therapeutic vulnerabilities.^6^, ^7^ Moreover, scRNA‐seq has proven advantageous in detecting and characterizing rare cell populations, such as cancer stem cells, which are often overlooked in bulk analysis.^6^, ^7^ By employing scRNA‐seq, investigations have been able to identify and explore these rare cell populations, opening new avenues for targeted therapies and providing deeper insights into their functional roles.^3^

Furthermore, scRNA‐seq serves as a powerful tool for capturing the dynamic transcriptional landscape of gastric tumours, enabling the study of cell state transitions and tumour progression.^3^, ^8^ It provides insights into the heterogeneity of GC and aids in the identification of potential therapeutic targets. Additionally, scRNA‐seq reveals distinct cell subpopulations associated with tumour characteristics and dysregulated signalling pathways.^4^ Moreover, it investigates the tumour microenvironment (TME) and its interactions with immune cells, furnishing valuable information for the development of immunotherapeutic targets.^5^

Despite the advancements in scRNA‐seq, challenges persist in its application to GC, primarily due to the high tumour heterogeneity and technical limitations. GCs require the characterization of diverse cell types, which presents obstacles such as cost, time and complexities in data analysis. Nevertheless, ongoing efforts are being made to refine scRNA‐seq protocols and develop computational tools tailored to GC research.^3^ This narrative review aims to explore the significance of scRNA‐seq in GCs by highlighting its advantages, addressing the challenges it faces and investigating its potential applications in understanding tumour heterogeneity and identifying therapeutic targets.

## 2 METHODOLOGY

This narrative review aims to present a comprehensive framework for evaluating the application of scRNA‐seq in GC research. To ensure a rigorous and inclusive approach, specific inclusion and exclusion criteria were employed.

The inclusion criteria for this review consisted of full‐text articles written in English, spanning from 2000 to 2023. This time period was chosen to allow for a thorough evaluation of established practices within the field as well as to capture any significant advancements that occurred over a substantial period of time. Multiple databases, including PubMed, EMBASE, Google Scholar, the Cochrane Library and Scopus, were employed to ensure a comprehensive literature review.

Key terms such as ‘scRNA Sequencing’ and ‘Single‐cell sequencing’ were used in combination with additional terms including ‘Gastric Cancer’, ‘Stomach tumours’, ‘Canceromics’, ‘tumour Microenvironment’ and ‘Cancer Heterogeneity’. This approach ensured that relevant articles addressing the intersection of scRNA‐seq and GC were included in the review.

In addition to the systematic database search, references cited in recent reviews focused on specific diseases were manually examined to identify additional sources that could contribute to the search strategy. Standalone abstracts, case reports, posters and unpublished or non‐peer‐reviewed studies were excluded. By adopting these exclusion criteria, the review aimed to prioritize the inclusion of high‐quality and reliable evidence.

Regarding the scope of the review, no predefined limit was set on the number of studies to be included. This approach was chosen to gather a comprehensive understanding of the subject matter and encompass a wide range of study designs. The review incorporated descriptive studies, animal‐model studies, cohort studies and observational studies, thereby providing a holistic perspective on the use of scRNA‐seq in GC research. The inclusion of investigations conducted in both pre‐clinical and clinical settings further contributed to the breadth of knowledge covered in this review. A summary of the methodology employed is presented in Table 1.

**TABLE 1:** Summary of methodology for this review.

Methodology steps	Description
Literature search	PubMed, EMBASE, Google Scholar, the Cochrane Library and Scopus
Inclusion criteria	Full‐text articles published in English Publication date range: 2000–2023 Focus on gastric cancer and gastric tumour
Exclusion criteria	Standalone abstracts Case reports Posters Unpublished or non‐peer‐reviewed studies
Search terms	Keywords such as ‘scRNA Sequencing’ and ‘Single‐cell sequencing’ coupled with indicators like ‘Gastric Cancer’, ‘Stomach Cancer’, ‘Tumour Heterogeneity’, ‘Tumour Microenvironment’, ‘Tumour Progression’, ‘Therapeutic Resistance’, ‘Canceromics’, ‘Tumour Microenvironment’ and ‘Cancer Heterogeneity’
Additional search	Manual examination of references cited in recent disease‐specific reviews No predetermined limit on the number of studies Encompassing diverse study designs: Descriptive studies Animal‐model studies Cohort studies Observational studies Including investigations in both pre‐clinical and clinical settings

## 3 RESULTS AND DISCUSSION

### 3.1 Types of ScRNA‐seq technology

ScRNA‐seq has revolutionized our understanding of GC by enabling the high‐resolution analysis of individual cells within the TME. Various scRNA‐seq technologies and platforms have been employed to dissect the complexity of GC at a single‐cell level. These platforms include droplet‐based methods such as 10× Genomics Chromium,^3^, ^8^, ^9^ Drop‐seq,^10^ inDrop,^11^ and protocols like Smart‐seq2,^3^, ^12^ each offering unique advantages in terms of throughput and transcriptome coverage. These platforms also include fluorescence‐activated cell sorting (FACS) methods such as Single‐Cell Signature Explorer,^13^ CEL‐seq,^14^ MARS‐seq,^15^ and SCI‐seq.^16^ These technologies have shed light on the heterogeneity of GC, revealing distinct cell populations, rare cell types and novel biomarkers. Furthermore, the integration of scRNA‐seq with other techniques, such as bulk RNA sequencing,^7^, ^17^ whole exome‐sequencing,^17^ machine learning algorithms such as ABSOLUTE and xCell,^18^ migration assay,^18^ or tumour spheroid assay,^18^ has provided a comprehensive view of both gene expression and cellular phenotypes in GC. As scRNA‐seq continues to evolve, it promises to drive the development of more precise diagnostic and therapeutic strategies for this complex disease. A summary of the types of scRNA‐seq technology is summarized in Table 2.

**TABLE 2:** Summary of the types of single cell RNA‐seq technology.

Platforms	Isolation strategies	Advantages	Disadvantages
Smart‐Seq2^3^, ^12^	FACS	Improves the capture of shorter transcripts Identifies transcriptional modifications such as gene shearing and allelic expression	Limited number of cells that could be processed
10× Genomics Chromium^3^, ^8^, ^9^	Microdroplets, FACS	High throughput sequencing platform More accurate evaluation of expression level, reducing quantitative bias	Not reported
Single‐Cell Signature Explorer^13^	FACS	High throughout signature exploration Comprehensive visualization of gene signatures	Not reported
Drop‐seq^10^	Microdroplets	High‐speed and highly parallel analysis of individual cells Reduction of time and cost in preparing libraries from many individual cells	Vulnerability to impurities
inDrop^11^	Microdroplets	High‐throughput sequencing platform	Single library‐type sequencing can result in low base composition diversity, leading to a spike in base call error rate
CEL‐seq^14^	FACS	High‐throughput sequencing platform Reproducible, linear and sensitive results	3′ bias and sensitivity to small copy numbers
MARS‐seq^15^	FACS	High‐throughput sequencing platform Spiked‐in technical controls show cell‐to‐cell variance compatible with sampling noise Improved information content of transcriptional states	Not reported
Seq‐Well^19^	Micro‐fluidic	High‐throughput sequencing platform Portable and low‐cost Efficient and suitable when working with limited samples	Not reported
SCI‐Seq^16^	FACS	High‐throughput sequencing platform Mitigates high library construction costs Does not require specialized microfluidics equipment or droplet emulsification techniques	Limited to the detection of copy‐number variants
Fluidigm C1^20^	Micro‐fluidic	Generation of high‐quality cDNA	Limitation of the cell capture process, leading to a large number of cells with false expression patterns
MATQ‐seq^21^	Micro‐fluidic	High accuracy and sensitivity Captures genuine biological variation between whole transciptomes of single cells	Not reported

Abbreviations: cDNA, complementary DNA; FACS, Fluorescence‐Activated Cell Sorting; MARS‐seq, Massively parallel single‐cell RNA; MATQ‐seq, Multiple Annealing and dc‐tailing‐Based Quantitative Single‐cell RNA‐Sequence.

### 3.2 Recent developments in ScRNA‐seq for gastric cancer research

#### 3.2.1 Cellular heterogeneity

GC is characterized by its high degree of heterogeneity, encompassing diverse malignant tumour populations.^7^ In recent years, the emergence of scRNA‐seq as a transformative approach has enabled comprehensive exploration of cellular heterogeneity, gene expression patterns and potential therapeutic targets in GC.

Using scRNA‐seq, hidden gene expression variations within GC cells have unveiled their molecular diversity. These patterns significantly affect GC progression, treatment responses and therapy resistance.^6^ Additionally, scRNA‐seq has uncovered diverse cell states in the GC microenvironment, encompassing immune cells, cancer stem cells and stromal components.^9^ Understanding these states is pivotal for deciphering GC mechanisms and refining targeted therapies. Tumour‐associated fibroblasts, altered by the tumour, form a supportive niche impacting GC growth and metastasis. scRNA‐seq has revealed their distinct subpopulations and functions.^7^ These findings have greatly advanced our understanding of disease progression and hold promise for targeted therapeutic interventions.^6^, ^7^, ^9^ Furthermore, scRNA‐seq has shed light on the existence of rare cancer stem cell‐like populations, which are often overlooked or diluted in bulk analyses. This knowledge improves our understanding of disease complexity and its challenging clinical prognosis.^3^ The insights derived from scRNA‐seq analyses are invaluable for the development of targeted therapies.

Moreover, scRNA‐seq provides unprecedented access to the dynamic transcriptional landscape of gastric tumours, facilitating the investigation of cellular transitions and the study of tumour progression. For instance, Deng et al. (2023) dissected the transcriptomic dynamics of GC progression in a murine model using scRNA‐seq, revealing distinct cellular states along the trajectory of disease advancement.^3^ In their study, they identified immunosuppressive subpopulations among tumour‐infiltrating immune cells and characterized cancer stem cells, shedding light on their roles in tumour growth and resistance.^3^ Similarly, Sun et al. (2022) employed scRNA‐seq to examine gastric tumours and unveiled a spectrum of cell states associated with disease progression.^8^ These seminal studies underscore the indispensable role of scRNA‐seq in capturing the temporal dynamics of tumour development and identifying crucial regulatory genes and pathways.

A comprehensive understanding of the TME is critical for delivering targeted and personalized therapies. Through scRNA‐seq and transcriptomic profiling, gastric tumour tissues have been found to harbour enriched populations of regulatory T cells (Tregs), characterized by heightened expression of immune suppression‐related genes, indicating an immunosuppressive TME.^22^ Additionally, the TME of GC lacks distinct exhausted clusters of differentiation 8 (CD8)+ T cells and exhibits low expression levels of exhaustion markers such as programmed cell death protein‐1 (PDCD1), cytotoxic T‐lymphocyte‐associated antigen 4 (CTLA4), Hepatitis A virus cellular receptor 2 (HAVCR2), lymphocyte‐activation gene 3 (LAG‐3) and T cell immunoreceptor with immunoglobulin and immunoreceptor tyrosine‐based inhibitory motif domains (TIGIT).^22^ Notably, the presence of atypical chemokine receptor 1 (ACKR1) in tumour endothelial cells has been linked to an unfavourable prognosis, while fibroblasts are implicated in tumour angiogenesis and the maintenance of tumour vasculature.^22^ These seminal discoveries elucidate the active cellular subtypes and their intricate interactions within the GC TME, substantially advancing our understanding of cellular heterogeneity. Similarly, scRNA‐seq analysis of GC, along with paired normal tissue and peripheral blood mononuclear cells (PBMC), has unravelled profound cellular deregulations within the TME, including stromal cells exhibiting distinct extracellular matrix (ECM) profiles compared to normal tissue, transcriptionally heterogeneous macrophages deviating from the conventional M1/M2 paradigm, unique gene expression programs of dendritic cells (DCs) in comparison to PBMC‐derived DCs, and exhausted cytotoxic T cells displaying two heterogeneous subsets.^23^ These findings collectively highlight the extensive reprogramming and cellular remodelling across multiple cellular elements in GC, manifesting as alterations in cell numbers, transcriptional states and intercellular interactions. Consequently, such insights hold immense potential for refined understanding and targeted personalized therapeutic strategies.^23^

Furthermore, scRNA‐seq has enabled the identification of prognostically independent subtypes of gastric adenocarcinoma (GAC) based on intratumoral heterogeneity, leading to the development of a 12‐gene prognostic signature.^6^ Additionally, scRNA‐seq analysis of various GC specimens, including differentiated GC (DGC), poorly differentiated GC (PDGC) and neuroendocrine carcinoma (NEC) has revealed the strong enrichment of PDGC in genes associated with the epithelial‐mesenchymal transition (EMT) program.^24^ Moreover, immune‐rich DGC tends to express genes responsive to interferon alpha and gamma, while immune‐poor PDGC exhibits no such tendency.^24^ During the transdifferentiation process from DGC to NEC, intermediate malignant cells display double‐negative expressions of DGC and NEC marker genes, accompanied by a gradual downregulation of interferon‐related pathways and decreased infiltration of CD8+ cytotoxic T cells.^24^ Similarly, integrative analysis of scRNA‐seq data from early gastric cardia adenocarcinoma (EGCA) and paired adjacent nonmalignant biopsy samples has unveiled the prevalence of gland and pit mucous cells, Aquaporin 5 (AQP5)+ stem cells, activated wingless‐related integration site (WNT) and nuclear factor kappa B (NF‐κB) signalling pathways, and increased nicotinamide N‐methyltransferase (NNMT) expression during malignant progression, all of which are associated with an unfavourable prognosis.^25^ These alterations in gene expression patterns provide valuable insights into the TME and potential therapeutic vulnerabilities that could be targeted in preventive medicine.

ScRNA‐seq has unravelled the intricate properties of cancer‐associated fibroblasts (CAFs) in GC. Through scRNA‐seq analyses, four CAF subsets with distinct characteristics have been identified, each exhibiting heightened protumour activities.^26^ Of particular interest are the inflammatory CAFs (iCAFs) and ECM CAFs (eCAFs), which engage in dynamic communication with adjacent immune cell subsets within the GC TME, promoting pro‐invasive activities and fostering a TME conducive to tumour growth.^26^ These findings reveal iCAFs and eCAFs as potential targets for therapeutic intervention.

Evaluation of human tissue samples using scRNA‐seq has further revealed the existence of malignant epithelial subclusters associated with invasive features, propensity for intraperitoneal metastasis, EMT‐induced tumour stem cell phenotypes and/or characteristics resembling dormancy within the TME.^27^ Moreover, high expression levels of genes associated with these subclusters have been correlated with poorer overall survival in GC patients.^27^

Finally, scRNA‐seq has provided valuable insights into the mechanisms underlying lymph node metastasis in GC through the analysis of primary GC tissues and paired metastatic lymph node cancer tissues. Individual cases exhibit distinct carcinoma profiles, diverse microenvironmental subsets and intratumoral heterogeneity. Furthermore, scRNA‐seq analysis has identified potential markers for GC lymph node metastasis, including erythroblastic oncogene B (ERBB) 2, claudin‐11 (CLDN11) and cyclin‐dependent kinase 12 (CDK12), as well as genes potentially driving the evolution of GC, such as Fos Proto‐Oncogene, AP‐1 Transcription Factor Subunit (FOS) and Jun Proto‐Oncogene, AP‐1 Transcription Factor Subunit (JUN).^12^ These findings offer crucial guidance for targeted therapy and inform preventive medicine strategies.

#### 3.2.2 Personalized therapeutic innovations: advancements and potential

The application of scRNA‐seq has revolutionized our understanding of disease mechanisms by uncovering gene expression alterations at the single‐cell level, providing valuable insights into the molecular landscape of diseases. In the context of gastric tumours, scRNA‐seq analysis has identified interleukin (IL) 17+ cells as potential therapeutic targets that influence tumour progression through IL17, IL22 and IL26 signalling pathways.^8^ Furthermore, scRNA‐seq studies of patient‐derived GC cells have revealed lineage‐specific drug sensitivities, such as the efficacy of vascular endothelial growth factor receptor (VEGFR) inhibitors for diffuse‐type tumours and protein kinase B (AKT) inhibition therapy for phosphatidylinositol‐4,5‐bisphosphate 3‐kinase catalytic subunit alpha (PIK3CA)‐E542K mutation.^28^ Network analysis of transcriptomic data has identified the transforming growth factor‐β (TGF‐β) pathway as a driver of mesenchymal behaviour in GC, which is associated with poor prognosis.^18^, ^29^

Moreover, scRNA‐seq has facilitated the development of a prognostic single‐patient classifier based on the activity of Granzyme B (GZMB), tryptophanyl‐tRNA synthetase (WARS) and secreted frizzled‐related protein 4 (SFRP4), leading to improved chemotherapy benefit prediction.^30^ Specific cell types contributing to tumour growth, such as iCAFs and eCAFs, have been identified through scRNA‐seq and could serve as potential therapeutic targets.^26^ Longitudinal scRNA‐seq analysis has revealed dynamic changes in the GC microenvironment post‐adjuvant chemotherapy, including alterations in immune cells, endothelial cells and proangiogenic pathways, highlighting the potential for personalized therapeutic strategies.^30^

Furthermore, scRNA‐seq analysis has uncovered genetic disparities and dysregulated gene expression in GC cells compared to normal tissue.^23^ The TME comprises various cell types, including stromal cells, macrophages, DCs and Tregs. Heterogeneous subpopulations have been observed within tumour‐associated DCs and cytotoxic T cells, along with the presence of immune checkpoints and co‐stimulatory molecules in immune cell subsets. Specific interactions between CAFs and GC cells have also been identified through scRNA‐seq.^23^ These findings provide insights into dynamic cellular changes in the GC microenvironment, uncovering potential targets for immunotherapy strategies.

#### 3.2.3 Exploring novel biomarkers for gastric cancer

The utilization of scRNA‐seq has enabled the discovery of clinical biomarkers in GCs, contributing to personalized treatment approaches. Wang, Zhang et al. (2021) identified FOS, JUN, ERBB2, CLDN11 and CDK12 as potential drivers of GC evolution, and Zhang et al. (2019) identified olfactory receptor family 51 subfamily E member 1 (OR51E1) and Hes Family basic helix–loop–helix transcription factor 6 (HES6) as biomarkers for early GC detection.^12^, ^31^ Moreover, Yang, Sun et al. (2022) found regulator of G protein signalling 2 (RGS2) to be a promising immunotherapy target, and Jiang et al. (2022) identified PD‐1 expression in CD8+ T cells as a predictive marker for treatment response.^27^, ^32^ Kang et al. (2022) also revealed M16‐C‐Type Lectin Domain Containing 9A (CLEC9A) DCs associated with prolonged survival, and EN10‐serpin family E member 1 (SERPINE1) endothelial cells linked to poor survival in GC patients.^17^

**FIGURE 1:**
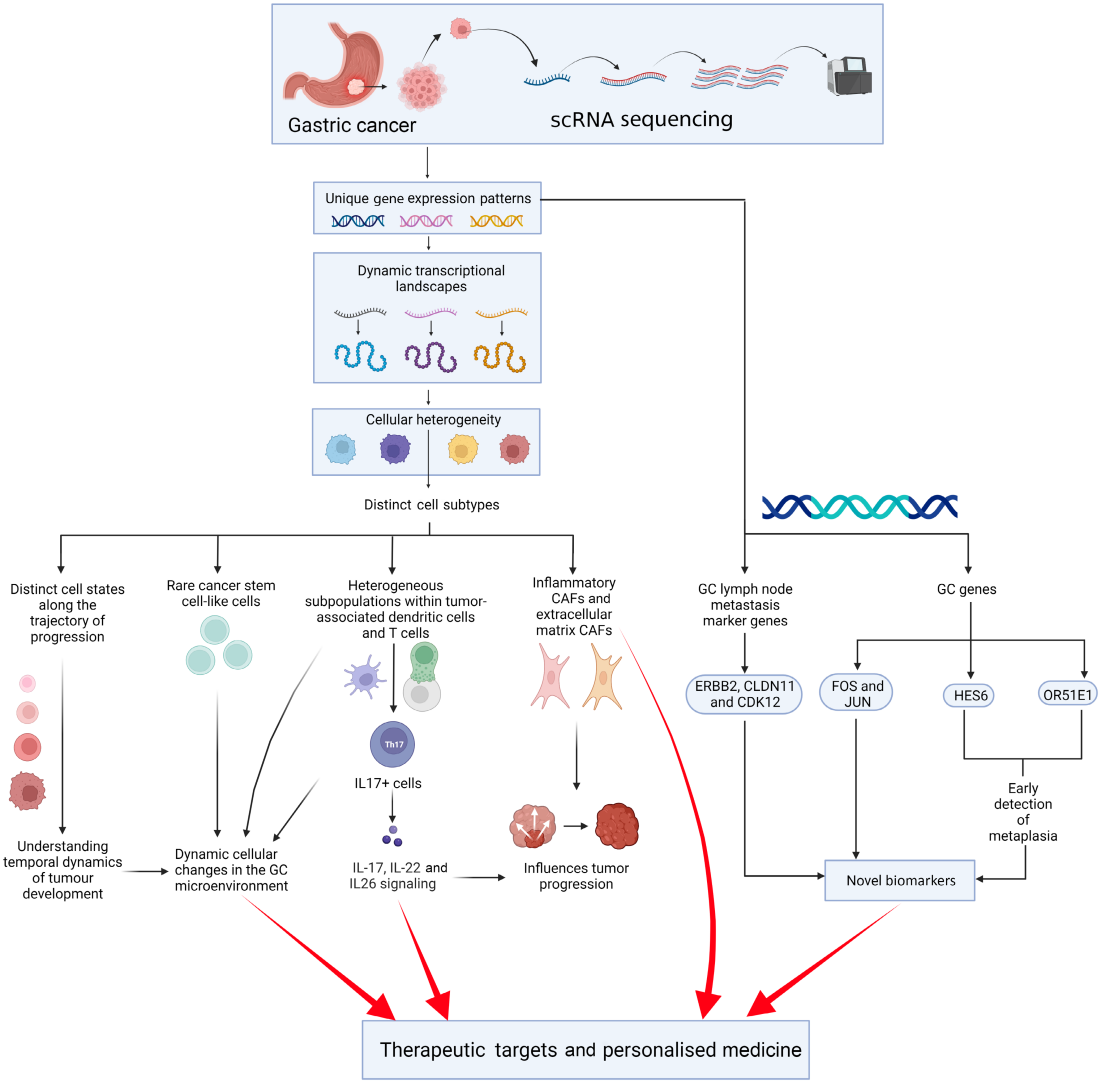
The recent developments in single‐cell RNA sequencing in gastric cancer research (created with Biorender.com). CDK12, cyclin‐dependent kinase 12; CLDN11, Claudin 11; ERBB2, Erythroblastic oncogene B 2; FOS, Fos Proto‐Oncogene AP‐1 Transcription Factor Subunit; GC, Gastric Cancer; HES6, Hes family basic helix–loop–helix transcription factor 6; IL, Interleukin; JUN, Jun Proto‐Oncogene; OR51E1, olfactory receptor family 51 subfamily E member 1; scRNA‐seq, single‐cell ribonucleic acid sequencing.

The integration of scRNA‐seq and spatial profiling data has highlighted the association of actin alpha 2 smooth muscle (ACTA2) expression with patient outcomes, while Liu et al. (2021) identified the metabolic enzymes methionine adenosyltransferase 2A (MAT2A) and adenosylhomocysteine hydrolase (AHCY) as potential targets for hepatoid adenocarcinoma treatment.^33^, ^34^ Additionally, Hu et al. (2022) identified tumour mutational burden, immune activation, and a high level of microsatellite instability (MSI‐H+) as favourable prognostic factors, and Yin et al. (2021) identified transition markers promoting GC development.^35^, ^36^ Notably, biglycan (BGN), cartilage oligomeric matrix protein (COMP), collagen type V alpha 2 chain (COL5A2) and secreted protein acidic and rich in cysteine (SPARC) were validated as diagnostic and prognostic indicators, while fatty acid‐binding protein 1 (FABP1) was linked to poor survival in GC patients.^37^, ^38^ These findings hold promise for targeted therapies and personalized medicine in GC. The recent developments in scRNA‐seq in GC have been summarized in Figure 1.

### 3.3 Challenges associated with scRNA‐seq in gastric cancer research

The utilization of scRNA‐seq has emerged as a potent tool for investigating the intricate cellular heterogeneity and underlying molecular processes in GCs. However, the application of scRNA‐seq is not without its challenges, which must be addressed to ensure accurate and reliable analysis.

#### 3.3.1 Low cell capture efficiency, sensitivity and depth of scRNA‐seq

A primary challenge in scRNA‐seq is achieving high cell capture efficiency, as this determines the completeness of the captured cellular landscape. Gastric tumours comprise diverse cell types, including tumour cells, stromal cells, immune cells and infiltrating lymphocytes. The inherent heterogeneity of these tumours complicates the accurate capture of all cell types, leading to potential bias and an incomplete understanding of the TME.^39^ Furthermore, the genetic mechanisms underlying familial and hereditary GCs, which constitute a small fraction of all cases, remain poorly understood. For example, only approximately one‐third of hereditary diffuse GC (HDGC) cases can be attributed to hereditary cadherin‐1 (CDH1) mutations among the various hereditary syndromes, such as GAC and proximal polyposis of the stomach (GAPPS) and familial intestinal GC (FIGC).^40^ The accurate identification and clustering of cell types are paramount for comprehending the cellular heterogeneity in gastric tumours.

The sensitivity and depth of scRNA‐seq are critical for detecting low‐abundance transcripts and quantifying gene expression levels accurately. In the context of gastric tumours, characterized by intricate cellular heterogeneity and transcriptomic changes, the limited sensitivity of scRNA‐seq poses challenges in identifying rare cell populations and subtle transcriptional variations.^3^ Despite the application of multi‐staining registration analysis in vitro, the isolation and enrichment of CAF within GCs remain arduous. Moreover, the dissociation process during scRNA‐seq has been observed to significantly affect the transcriptome of cells, leading to suboptimal yields of specific cell subsets.^26^ This can hinder the accurate identification of heterogeneity in various cell types, including T and natural killer (NK) cells, myeloid cells, fibroblasts and endothelial cells within GCs.^22^

A low sequencing depth in scRNA‐seq presents challenges in detecting lowly expressed genes and alternative splicing events.^31^ Furthermore, certain cell types, such as granulocytes, prove challenging to target due to their low RNA content and high levels of ribonuclease (RNase).^41^ Collectively, these challenges contribute to the complexities and limitations associated with scRNA‐seq analysis in gastric tumours.

#### 3.3.2 Interpreting cell states and lineages in scRNA‐seq data

Interpreting cell states and lineages within GCs poses a significant challenge in scRNA‐seq analysis. These tumours exhibit intricate cellular heterogeneity with diverse subtypes and differentiation states. However, identifying and characterizing distinct cell populations becomes challenging due to the dynamic nature of gene expression patterns and the potential presence of transitional cell states.^27^ Additionally, scRNA‐seq fails to account for vital information on the spatial distribution and chromatin accessibility of distinct cell types, resulting in a loss of spatial origin at the individual cell level.^8^ The tissue dissociation techniques employed in scRNA‐seq further contribute to this loss of spatial information.^31^ Consequently, interpreting cell states and lineages becomes intricate. Moreover, the lack of analytical methods for multi‐omics data impedes the comprehensive interpretation of cell states and lineages.^42^ As a result, the complexity increases when trying to understand cell states and lineages, while the absence of analytical techniques for multi‐omics data further hinders the comprehensive interpretation of these cellular phenomena.^42^

#### 3.3.3 Financial and ethical considerations in scRNA‐seq studies

Implementing scRNA‐seq in research and clinical settings encounters notable financial and ethical challenges. The high costs associated with acquiring scRNA‐seq platforms, such as the 10× Genomics Chromium system or the Fluidigm C1 system, pose a barrier for many institutions.^31^ Additionally, recurring expenses for consumables, reagents and sequencing services strain research budgets, limiting the scale of GC heterogeneity studies.^43^

The analysis phase of scRNA‐seq data presents further financial burdens. Computational resources, including high‐performance computing clusters and specialized bioinformatics pipelines, are essential but costly.^44^ Establishing and maintaining such infrastructure and needing bioinformatics expertise increases institutions' financial strain.^45^ Accessible and cost‐effective analysis pipelines and resources are necessary to promote widespread and equitable use of scRNA‐seq in GC research.

Ethical challenges arise due to the nature of scRNA‐seq data and potential privacy risks. Obtaining informed consent from patients, especially when deconvoluting individual information from aggregated datasets, is complex.^8^ Protecting patient privacy through robust data anonymization strategies and adherence to ethical guidelines, such as the Health Insurance Portability and Accountability Act (HIPAA) Privacy Rule is crucial. Standardized consent procedures and frameworks tailored to scRNA‐seq in GCs can ensure the responsible use of patient‐derived data.^46^

**FIGURE 2:**
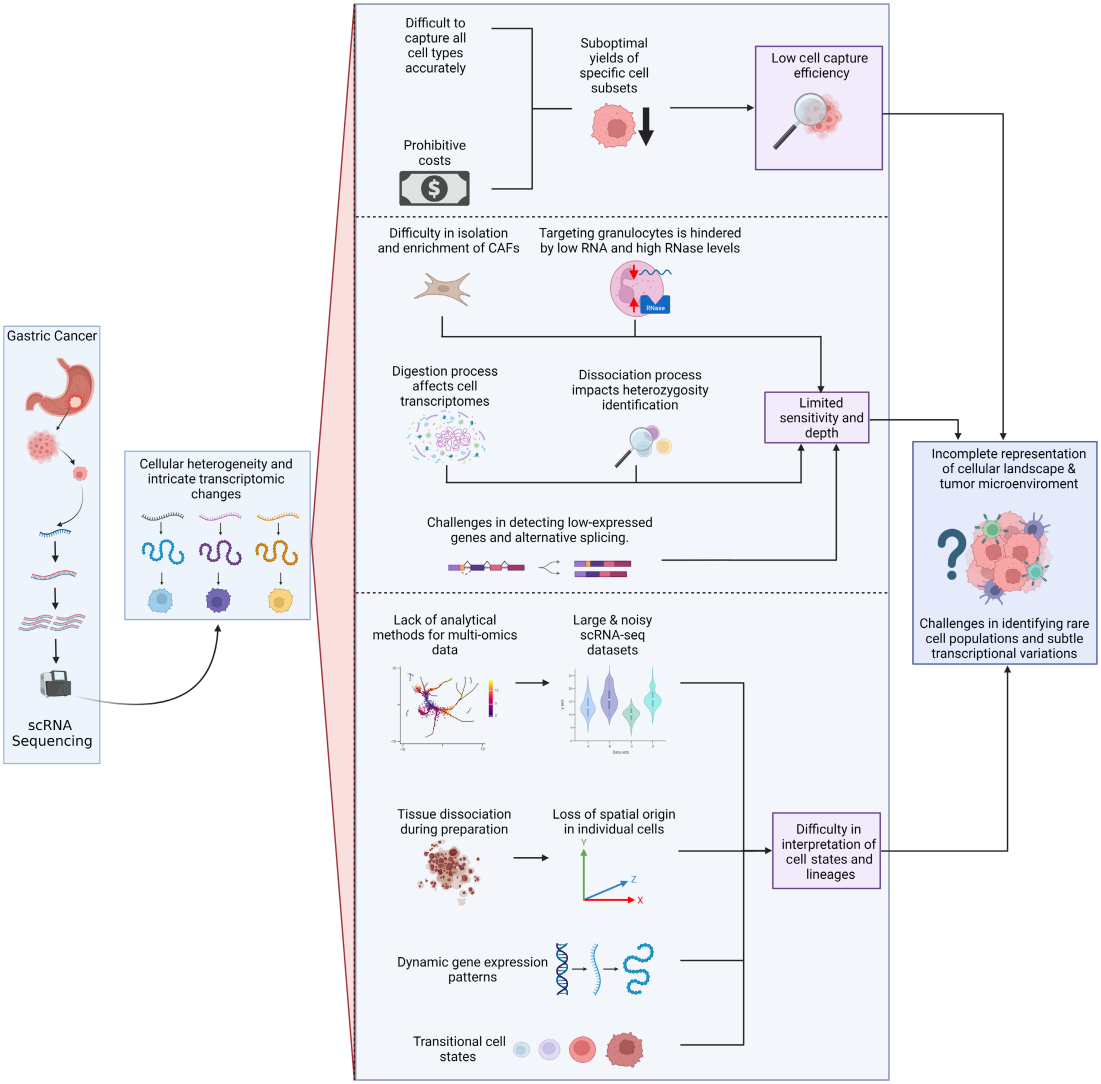
Challenges associated with single‐cell RNA‐sequencing in gastric cancer research (Created with Biorender.com). CAFs, cancer‐associated fibroblasts; RNA, ribonucleic acid; RNase, ribonuclease; scRNA‐seq, single‐cell ribonucleic acid sequencing.

Data sharing and intellectual property pose ethical challenges. While data sharing promotes collaboration and knowledge advancement, concerns about intellectual property rights and commercial interests hinder open access to scRNA‐seq datasets.^9^ The challenges associated with scRNA‐seq in GC research have been illustrated in Figure 2.

### 3.4 Ongoing efforts to overcome challenges and improve scRNA‐seq techniques in gastric cancer research

#### 3.4.1 Applications of machine learning in scRNA‐seq

The field of genomic oncology has witnessed a transformative impact with the advent of scRNA‐seq, enabling gene expression profiling at the cellular level. However, the analysis of scRNA‐seq data still presents challenges such as high dimensionality, sparsity and technical noise. In response, machine learning (ML) and deep learning (DL) techniques have emerged as valuable tools for automated and objective analysis.

Ensemble‐based algorithms, including Random Forest, have demonstrated promising results in predicting Helicobacter pylori positivity in GC samples, achieving 97% accuracy.^47^ The scPred method, using dimensionality reduction and support vector machine classification, accurately identified tumour epithelial cells in GC with an area under the receiver operating characteristic (AUROC) of 0.999.^48^ ML algorithms employing uniform manifold approximation and projection (UMAP) identified 10 immune‐related gene signatures with superior prognostic prediction performance in GC patients.^49^ Moreover, the gene VCAN emerged as a potential predictor for response to immune checkpoint blockade therapies using the least absolute shrinkage and selection operator (LASSO) method.^50^

DL techniques, which leverage artificial neural networks, were effective in distinguishing GC subtypes through autoencoder‐based weight initialisation.^51^ Deep generative models, such as generative adversarial networks, facilitated pattern discovery and improved downstream analysis by generating synthetic scRNA‐seq data.^23^ However, it is critical to further investigate the generalisability of ML models, as their performance varied when validated with clinical data from the STAD‐TCGA dataset.^32^

ML approaches also contributed to the development of a fatty acid prognostic risk model (FARS), which showed promising prognostic efficacy. Through single‐cell analysis, differentially expressed genes and fatty acid metabolism in gastric tumour tissues were analysed, leading to the discovery of a novel biomarker, the RGS2 gene.^32^ These ML methods have proven valuable in predicting treatment response, identifying prognostic markers and uncovering biological insights, offering the potential for improving tumour prognosis and clinical decision‐making.

#### 3.4.2 Development of other novel computational tools to enhance scRNA‐seq in gastric cancer

Continuous advancements in computational methods are essential for the accurate analysis of scRNA‐seq data in GCs. Efforts are focused on developing algorithms and tools to address challenges such as batch effects, cell type identification, trajectory inference and low cell capture efficiency.

To improve cell capture efficiency, techniques such as cellular indexing of transcriptomes and epitopes (CITE‐seq) have been introduced. This technique utilizes streptavidin‐biotin interactions to link the 5′ end of oligos to antibodies, enabling the analysis of transcriptomes alongside cell surface protein abundance at the single‐cell level.^39^ The addition of a dithiothreitol (DTT) buffer offers the opportunity to produce DNAs with high purity, aiding the combined analysis of genetic information and protein abundance.^39^ Additionally, modified scRNA‐seq protocols incorporating unique molecular identifiers (UMIs) and droplet‐based barcoding.^3^ This technique prevents erroneous repeated counting of each reverse transcription product, thereby have improving sensitivity and accuracy in profiling gastric tumour cells.^3^

Pseudo‐time analysis and trajectory inference algorithms have played important roles in understanding the progression and cellular dynamics of gastric tumours.^32^ Spatial transcriptomics and single‐cell assays for transposase‐accessible chromatin sequencing (scATAC‐seq) provide valuable spatial context and information on transcription factors (TFs).^8^ Computational tools like Harmony have been developed to address batch effects and enable the integration and comparison of scRNA‐seq datasets from different experiments or platforms.^37^ Integration with other ‐omics data, such as single‐cell DNA sequencing and epigenetic profiling, further enhances resolution and provides comprehensive insights into genetic and epigenetic alterations in gastric tumours.^7^

**FIGURE 3:**
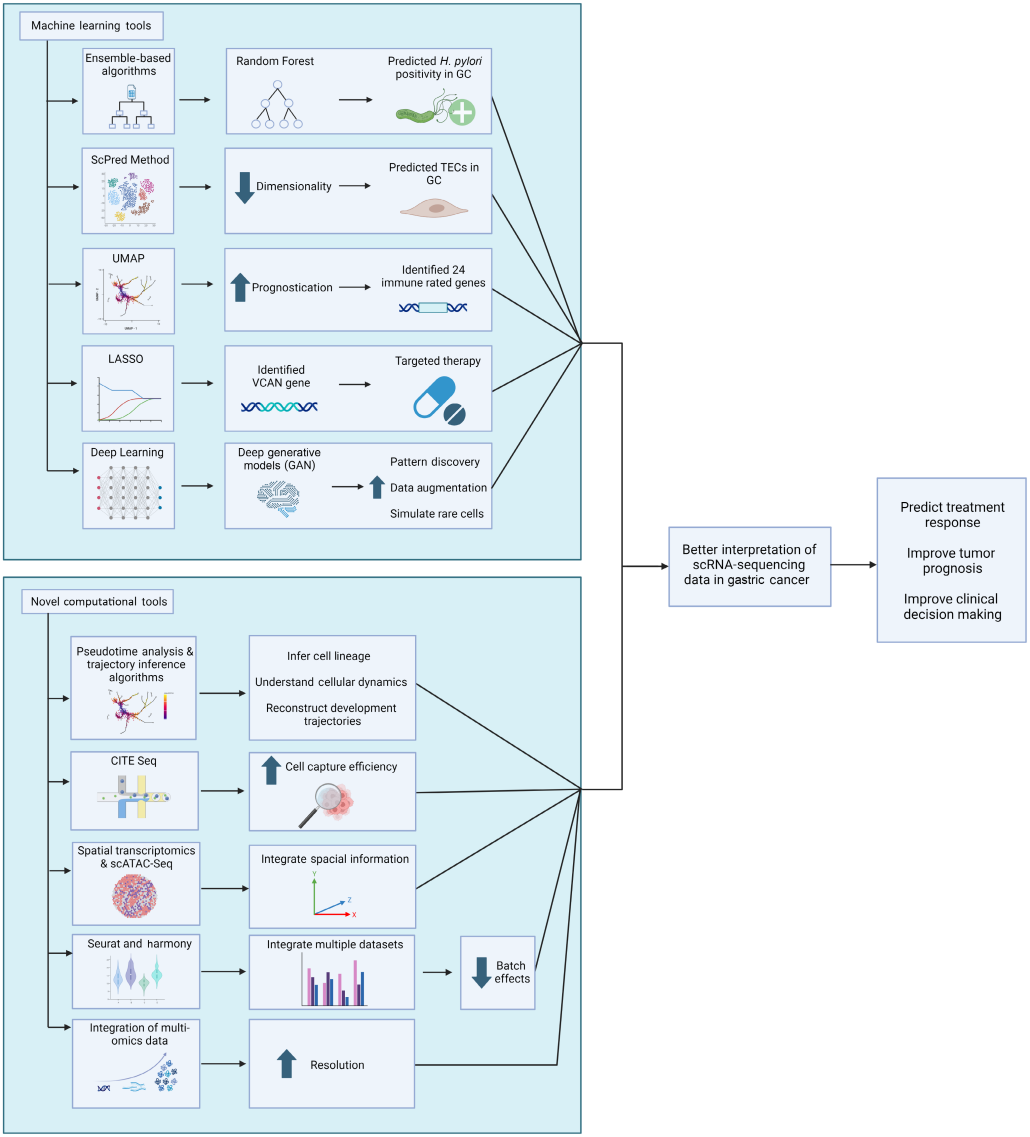
Recent Efforts to overcome the challenges in single‐cell RNA‐sequencing in gastric cancer research. CITE, cellular indexing of transcriptomes and epitopes; GC, gastric cancer; LASSO, least absolute shrinkage and selection operator; scATAC‐Seq, single‐cell assays for transposase‐accessible chromatin sequencing; scRNA‐seq, single‐cell ribonucleic acid sequencing; UMAP, uniform manifold approximation and projection.

Efforts to mitigate batch effects and technical variability involve batch correction algorithms like Seurat and Harmony, quality control metrics and normalization methods.^52^ Accurate cell type identification remains a challenge, with computational frameworks like CellAssign improving identification accuracy by integrating scRNA‐seq data with marker genes.^53^ These computational advancements contribute to obtaining robust and biologically meaningful insights from scRNA‐seq studies in GCs. Efforts to overcome the scRNA‐seq studies in GCs have been illustrated in Figure 3.

#### 3.4.3 Improving cost and accessibility

In recent years, there have been significant advancements in both the accessibility and affordability of scRNA‐Seq technologies. These improvements have played a pivotal role in facilitating the widespread adoption of scRNA‐Seq in GC research. For instance, in 2005, the US National Institutes of Health launched the Cancer Genome Atlas (TCGA), a groundbreaking pilot project with a substantial budget of $100 million. This initiative marked the inception of comprehensive genomic profiling in cancer research, encompassing various cancer types, including GC.^54^

Simultaneously, the International Cancer Genome Consortium (ICGC) embarked on a global mission to characterize genomic alterations in diverse cancers. These groundbreaking initiatives served as the cornerstone for subsequent endeavours aimed at reducing costs and enhancing accessibility to scRNA‐Seq technologies in low‐resource settings.^55^ Furthermore, inspired by the success of TCGA and ICGC, collaborations and data‐sharing initiatives emerged, contributing to the availability of invaluable scRNA‐Seq datasets. These collaborative efforts have further improved accessibility for researchers engaged in GC and other malignancy studies.^54^, ^55^

Lastly, the Human Genome Sequencing Project heightened the scientific community's awareness of the potential for cost reductions through advancements in sequencing technologies. This vision materialized with the emergence of massive parallel sequencing technologies, effectively dismantling the barriers that had previously limited access to scRNA‐Seq research.^56^

### 3.5 Future perspectives and outlook

The potential of scRNA‐seq to advance our knowledge of GC is significant, despite the current challenges it presents. scRNA‐seq offers promising benefits and prospects for GC research, particularly in understanding the dynamic nature of tumour evolution and progression.^57^ By enabling the tracking of clonal evolution, identification of driver mutations and examination of cellular phenotypic transitions, scRNA‐seq provides valuable insights into the underlying mechanisms driving GC progression, thereby guiding the development of more effective therapeutic strategies.

To fully exploit the potential of scRNA‐seq, certain advancements are required. Firstly, there is a need to develop more efficient and cost‐effective technologies or protocols for specimen preparation and sequencing. These advancements would increase the scalability and accessibility of scRNA‐seq, particularly in low‐ and middle‐income countries.^39^ Additionally, future research should focus on creating analytical approaches that integrate scRNA‐seq with other omics data, such as genomics and epigenomics, to comprehensively interpret cell states and lineages.^42^ This multi‐omics technique would significantly contribute to our understanding of GC biology.

Financial considerations play an important role in assessing the feasibility and sustainability of implementing scRNA‐seq. Currently, the costs associated with novel scRNA‐seq technology are higher than those of bulk RNA‐seq for GC patients.^3^ Future studies should explore more cost‐effective methods of using scRNA‐seq. Lessons can be learned from other technologies, as subsequent studies indicate potential cost reductions with technological advancements, including the use of high‐throughput sequencing platforms and improved protocols.^58^

Ethical concerns, particularly in human subject research, are of paramount importance. As such, data about the developmental potential and hierarchical relationships among early human haematopoietic progenitors is well‐protected.^59^ However, scRNA‐seq enables date mapping and lineage tracing. This could raise potential ethical concerns due to an unintentional breach of privacy.^59^ Prioritizing this aspect promotes ethical practice of genomic medicine.

Lastly, conducting larger‐scale scRNA‐seq cohorts encompassing diverse populations from different regions and income levels will enhance the representativeness and generalisability of findings. This comprehensive approach ensures a deeper understanding of GC by accounting for population variations.

## 4 CONCLUSION

In conclusion, this review highlights the transformative nature of scRNA‐seq in GC research. By unravelling cellular heterogeneity, gene expression dynamics and interactions within the TME, scRNA‐seq provides invaluable insights into tumour progression and potential therapeutic targets. This technology allows for the identification of distinct cell populations, rare cells and dynamic transcriptional changes, which contribute to our understanding of GCs.

Moreover, scRNA‐seq has the potential to uncover novel biomarkers and personalized treatment strategies, improving patient care. Although challenges remain, including protocol standardization and computational analysis, recent advancements in these areas offer promise for overcoming limitations and further advancing our knowledge of GCs.

The significant impact of scRNA‐seq in biomedical research, along with its potential to improve patient outcomes, requires further exploration and the resolution of challenges to fully realize its transformative potential in medicine. Continued efforts in this field will undoubtedly contribute to advancements in GC research and pave the way for more precise and effective therapeutic interventions.

## AUTHOR CONTRIBUTIONS

**Wireko Andrew Awuah:** Conceptualization (equal); data curation (equal); formal analysis (equal); investigation (equal); methodology (equal); project administration (equal); supervision (equal); writing – original draft (equal); writing – review and editing (equal). **Sakshi Roy:** Conceptualization (equal); data curation (equal); formal analysis (equal); investigation (equal); methodology (equal); project administration (equal); software (equal); supervision (equal); validation (equal); visualization (equal); writing – original draft (equal); writing – review and editing (equal). **Joecelyn Kirani Tan:** Data curation (equal); methodology (equal); validation (equal); writing – original draft (equal); writing – review and editing (equal). **Favour Tope Adebusoye:** Data curation (equal); methodology (equal); validation (equal); writing – original draft (equal); writing – review and editing (equal). **Zekai Qiang:** Data curation (equal); methodology (equal); validation (equal); writing – original draft (equal); writing – review and editing (equal). **Tomas Ferreira:** Data curation (equal); methodology (equal); validation (equal); writing – original draft (equal); writing – review and editing (equal). **Arjun Ahluwalia:** Data curation (equal); methodology (equal); validation (equal); writing – original draft (equal); writing – review and editing (equal). **Vallabh Shet:** Data curation (equal); methodology (equal); validation (equal); writing – original draft (equal); writing – review and editing (equal). **Amanda Leong Weng Yee:** Data curation (equal); methodology (equal); validation (equal); writing – original draft (equal); writing – review and editing (equal). **Toufik Abdul‐Rahman:** Data curation (equal); methodology (equal); validation (equal); writing – original draft (equal); writing – review and editing (equal). **Marios Papadakis:** Data curation (equal); methodology (equal); validation (equal); writing – original draft (equal); writing – review and editing (equal).

## FUNDING INFORMATION

The authors declare that no funds, grants or other support were received during the preparation of this manuscript.

## CONFLICT OF INTEREST STATEMENT

The authors have no relevant financial or non‐financial interests to disclose.

## CONSENT TO PARTICIPATE

No original data from new patients were collected, consent to participate is not applicable.
